# Organ donor pancreases for the study of human islet cell histology and pathophysiology: a precious and valuable resource

**DOI:** 10.1007/s00125-018-4546-x

**Published:** 2018-01-21

**Authors:** Piero Marchetti, Mara Suleiman, Lorella Marselli

**Affiliations:** Department of Clinical and Experimental Medicine and University Hospital, University of Pisa, via Paradisa 2, 56121 Pisa, Italy

**Keywords:** Autoptic samples, Beta cells, Islet cells, Organ donors, Pancreatectomy, Pancreatic biopsies

## Abstract

Direct in vivo assessment of pancreatic islet-cells for the study of the pathophysiology of diabetes in humans is hampered by anatomical and technological hurdles. To date, most of the information that has been generated is derived from histological studies performed on pancreatic tissue from autopsy, surgery, in vivo biopsy or organ donation. Each approach has its advantages and disadvantages (as summarised in this commentary); however, in this edition of *Diabetologia*, Kusmartseva et al (10.1007/s00125-017-4494-x) provide further evidence to support the use of organ donor pancreases for the study of human diabetes. They show that length of terminal hospitalisation of organ donors prior to death does not seem to influence the frequency of inflammatory cells infiltrating the pancreas and the replication of beta cells. These findings are reassuring, demonstrating the reliability of this precious and valuable resource for human islet cells research.

## Introduction

Insulin-secreting beta cells, sophisticated and unique endocrine cells, comprise the majority of cells in the pancreatic islets. Their failure, owing to the interplay of genetic, epigenetic and environmental factors, is key to the onset and progression of type 1 and type 2 diabetes [1, 2]. Therefore, there is much interest in investigating the properties of beta cells in health and disease, with the hope of improving strategies for diabetes prevention and treatment [1–4]. However, direct evaluation of beta cell features in vivo is substantially impeded by several factors. For instance, the pancreas is located deep in the abdomen, in close vicinity and/or direct contact to vital organs and vessels, and this anatomy makes accessibility extremely limited. In addition, despite much ongoing work, clinical-grade contrast agents and suitable techniques for beta cell imaging are not currently available [5]. Further, owing to the relatively small size of islets, their scattered distribution in the pancreas and the low total amount of beta cells (≤1–2% of the whole pancreatic mass), it is difficult to study this organ. As a consequence, most information on islet and beta cell pathophysiology in humans derives from histological studies.

## Sources of pancreatic tissue

### Autopsy samples

For over a century since the discovery of the pancreatic islets in the rabbit by Paul Langerhans [6], microscopy-based investigations of the human pancreas have used samples from organs obtained at autopsy [7]. This work has resulted in pioneering observations, such as the discovery of lesions in islets from individuals with diabetes, which was the first evidence for a link between alterations in islet morphology and the disease [8]. Following on, particularly in the past few decades, changes in islet and/or beta cell mass, volume and area have been reported in autoptic pancreatic samples of those with either type 1 and (although with less consistent results) type 2 diabetes [9–12]. Such an approach has also allowed for the assessment of morphological patterns associated with beta cell death and, in some cases, beta cell regeneration [9–12]. One advantage of using pancreases from autopsies is the possibility to access the whole gland, which may permit detailed morphometric studies. However, the quality of autoptic pancreatic specimens may be jeopardised by variability in cold ischaemia time (CIT; the time between organ removal and chilling of the tissue), which in turn could lead to tissue autolysis and, therefore, limit the morphological quality of the samples and the stability of their cellular components [13].

### Samples from surgery

More recently, pancreatic surgical samples obtained after removal of portions of the gland (usually owing to the presence of pancreatic tumours) have been used in research. This approach has been generally applied for the comparison of islets and beta cells from those with and without type 2 diabetes, and also to study beta cell traits in individuals with insulin resistance [14–17]. Obvious advantages associated with the use of surgical samples are, for instance, minimal cold ischaemia and the possibility to evaluate insulin secretion and insulin sensitivity in the patients before and even after pancreatectomy, for analysis of correlation with histological findings. In addition, techniques have been developed for the in situ study of pancreatic exocrine and endocrine cells, using tissue slides from surgical pancreas samples [18]. At the same time, a number of limitations should be taken into account, such as the possible presence of diabetes caused by the underlying disorder of the exocrine pancreas (type 3c diabetes, which may be erroneously classified as type 2 diabetes) [19, 20], the limited amount of tissue yielded, the variable regions of the pancreas subjected to resection and the possible influence of the diverse tumours on neighbouring cells.

### Laparoscopic-assisted biopsy samples

On a few occasions, pancreatic tissue samples have been obtained by laparoscopic-assisted biopsy procedures in individuals with recent-onset type 1 diabetes [21–23]. This has allowed for the study of several morphological, functional and molecular properties of the islets and the beta cells early in the natural history of the disease, in samples of very good quality. However, pancreatic biopsy carries a high risk of complications, such as postoperative bleeding and leakage of amylase-rich pancreatic juice from the margins of the resection, which makes the use of this procedure for research purposes unacceptable in most cases.

### Brain-dead organ donation

**Fig. 1:**
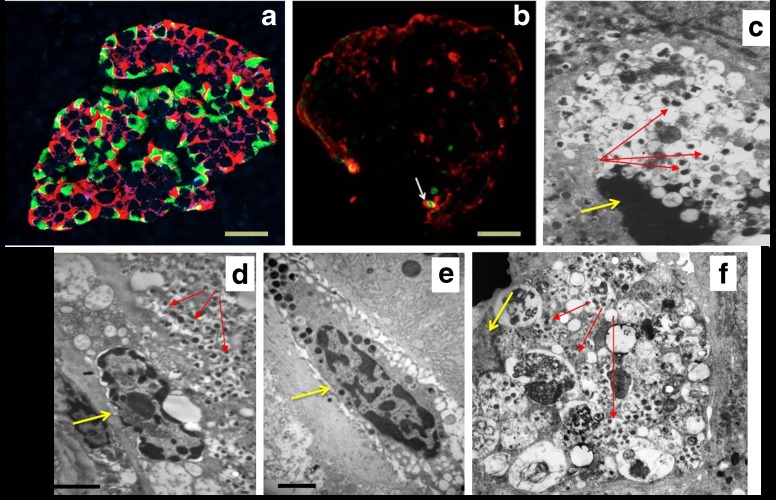
Representative images, obtained by light or electron microscopy, of pancreatic samples yielded from organ donors. (**a**) Immunofluorescence analysis of an islet from a donor without diabetes; insulin containing cells are in red, glucagon containing cells are in green (M. Suleiman, personal communication). (**b**) Immunofluorescence analysis of an islet containing a beta cell with an apoptotic nucleus (white arrow); insulin containing cells are in red, the apoptotic nucleus is in green (TUNEL staining). Scale bars in (**a**) and (**b**), 20 μm. (**c**) An apoptotic beta cell visualised by electron microscopy; the yellow arrow indicates an apoptotic nucleus with marked chromatin condensation, the red arrows indicate insulin granules. Magnification ×10,000. (**d**, **e**) Electron micrograph showing immune cells infiltrating the pancreas. In (**d**), the yellow arrows indicate a macrophage and red arrows indicate insulin granules in a beta cell adjacent to the macrophage. In (**e**) the yellow arrow points to a mast cell. Scale bars in (**d**) and (**e**), 1 μm. (**f**) Electron micrograph showing a beta cell with signs of death associated with altered autophagy (note the cytoplasm engulfed with lysosomes [multigranular bodies]); the yellow arrow indicates the nucleus without evidence of apoptotic features, the red arrows point to some remaining insulin granules. Magnification ×7000. Figure parts (**b**), (**d**) and (**e**) adapted from [29] with permission from Springer Nature, copyright 2015; figure parts (**c**) and (**f**) adapted from [31] with permission from Springer Nature, copyright 2009

Many researchers have started to use whole pancreases (or part of them) retrieved from brain-dead multi-organ donors. The advantages of using such a resource include the transplantation-grade quality of the tissue, better representation of the demographics of the general population, and possible availability of additional tissues, such as spleen and pancreatic/non-pancreatic lymph nodes [13]. Indeed, islet density, beta cell amount and turnover and beta cell ultrastructure have been evaluated using donor pancreatic samples from both non-diabetic and diabetic individuals (Fig. 1a–c, f), together with the assessment of innate and adaptive immune cell presence in the islets (which is presumed to be implicated in beta cell dysfunction and death) (Fig. 1d, e) [24–30]. Further, islets isolated by enzymatic digestion or acquired by laser capture microdissection may be obtained from the same glands that are used for histology and can, therefore, be characterised in terms of functional, ultrastructural and molecular features for association studies [31, 32].

## Validity of brain-dead organ donor pancreases for research

For the reasons mentioned above, the use of organ donor pancreases in research is being extensively implemented by single research groups and also, very importantly, by dedicated networks. For example, the Network for Pancreatic Organ donors with Diabetes (nPOD; www.jdrfnpod.org), which was started in 2007 by JDRF in the USA, has reported use of organ donor pancreases in their research. Similarly, the more recent programme INNODIA (an Innovative Medicines Initiative [IMI] 2 Joint Undertaking, receiving support from the European Union’s Horizon 2020 research and innovation programme and the European Federation of Pharmaceutical Industries and Associations [EFPIA], JDRF, and the Leona M. and Harry B. Helmsley Charitable Trust; https://innodia.eu/) has also adopted use of these samples. However, there are some concerns regarding the use of organ donor pancreases. For instance, the longer the interval between organ removal and tissue processing for clinical or research purposes (CIT), the worse the outcome of pancreas transplant or islet isolation [33]. However, the duration of CIT can be controlled by the operators and is consistently registered, which should avoid the use of organs of poor quality caused by excessive CIT duration. On the contrary, for obvious reasons, research teams have no control of other typical steps in the organ donation timeline, such as the duration of hospital admission prior to brain death and the brain death to organ removal interval, both of which have been suggested to affect the properties of pancreatic non-endocrine and endocrine cells.

Importantly, however, in this edition of *Diabetologia*, Kusmartseva et al [34] report that duration of hospital time prior to organ donor death does not affect the frequency of cell infiltration into the pancreas or the amount of replicating beta cells, thus reinforcing the validity and value of this precious resource. The authors studied pancreases from 39 organ donors without a history of diabetes and collected sample sections from tissue blocks of the different regions (head, body and tail) of the gland. The donors were age, sex, BMI and ethnicity-matched in groups of three, and within each group, individuals varied in terms of duration of hospitalisation stay prior to organ retrieval (<3 days, 3 to <6 days or ≥6 days). In addition, the authors gathered information on the medications administered prior to death. Single or double immunohistochemistry staining was then performed to identify cells positive for insulin, the leucocyte marker CD45, the macrophage marker CD68 and the replication marker Ki67. The authors found that there were no differences in CD45^+^ and CD68^+^ cell frequencies between hospitalisation groups, suggesting no major impact of hospitalisation stay on the amount of these immune cells in the pancreas. In addition, the interaction of hospitalisation time with age group of organ donors also did not influence the amount of infiltrating immune cells. However, they observed that the frequency of CD45^+^ cells was lower in the head and the body vs tail areas. These regional differences have not been taken into account in a previous study addressing similar issues [35], which interestingly reported that a significantly higher infiltration of CD45^+^ and CD68^+^ in the body portion of the pancreas from organ donors, accompanied prolonged life support (the head and tail were not examined in this report).

Kusmartseva et al also found that length of hospitalisation prior to organ donation did not significantly influence beta cell replication, as assessed by evaluating the presence of the marker Ki67 in insulin positive cells. Cells positive for both insulin and Ki67 staining have been previously observed in surgical pancreatic samples [36] and some (although not all) studies using samples from organ donor pancreases [35, 36]. Interestingly, analyses with autoptic pancreatic specimens have shown extremely low or absent amounts of ‘replicating’ beta cells, suggesting that such a finding could, in part, be an artefact of the postmortem state [37].

## Conclusions

In conclusion, whilst each source of pancreatic tissue from humans has advantages and disadvantages (summarised in Table 1), evidence is accumulating, showing that organ donor pancreases are a precious and valuable resource for the study of the histology and pathophysiology of human islet cells. Although the process from donor hospital admission to pancreas retrieval cannot always be fully controlled, the duration of hospital stay until brain death (a major step in the organ donation timeline), does not seem to be associated with significant changes in key histological features of the retrieved pancreas, such as leucocyte and macrophage infiltration, and frequency of replicating beta cells. In addition, in a previous study, duration of final hospitalisation was shown not to be negatively associated with the quality of nucleic acid extracted from organ donor pancreases [38]. However, it must be noted that all the studies that have investigated the correlation between hospital time before brain death and pancreatic histopathological features have been conducted using tissue from donors without diabetes and it is not known whether similarly encouraging findings are applicable to organ donors with diabetes. Furthermore, it must be taken into account that little information, if any, is available on the histology of the human pancreas should the gland come from donors after cardiac death (i.e. with permanent cessation of circulatory and respiratory functions) [39, 40]. In Europe [41] and in the USA [42] there are around 10,000 deceased organ donors per year and only 10–20% of the available pancreases are used for clinical transplantation. More effort should therefore be made to implement the use of donor pancreases for research purposes.

**Table 1:** Main advantages and disadvantages of pancreas sources

Source	Advantages	Disadvantages	Overall tissue quality
Autopsy	Access to the whole pancreas Use of autoptic tissue banks	Possible postmortem artefacts	Fair to good
Surgery	Minimal CIT Phenotypic characterisation of patients undergoing surgery	Limited amount of tissue Type 3c diabetes to be considered Possible influences of tumour on islet cell traits	Very good
Biopsy	The pancreatic tissue source closest to the in vivo situation	Limited amount of tissue Complications owing to the procedure (bleeding, leakage)	Excellent
Organ donation	Transplantation-grade procedures Possibility to prepare isolated islets	Limited information on family and clinical history Limited researcher control of steps from donor hospital admission to tissue processing	Very good

## Abbreviation

CIT: Cold ischaemia time
